# Plant SWEETs: from sugar transport to plant–pathogen interaction and more unexpected physiological roles

**DOI:** 10.1093/plphys/kiab127

**Published:** 2021-03-16

**Authors:** Richard Breia, Artur Conde, Hélder Badim, Ana Margarida Fortes, Hernâni Gerós, Antonio Granell

**Affiliations:** 1 Centre of Molecular and Environmental Biology (CBMA), Department of Biology, University of Minho, Braga 4710-057, Portugal; 2 Centre for the Research and Technology of Agro-Environmental and Biological Sciences (CITAB), University of Trás-os-Montes e Alto Douro, Vila Real 5001-801, Portugal; 3 Lisbon Science Faculty, BioISI, University of Lisbon, Campo Grande, Lisbon 1749-016, Portugal; 4 Centre of Biological Engineering (CEB), Department of Engineering, University of Minho, Braga 4710-057, Portugal; 5 Institute of Molecular and Cellular Biology of Plants, Spanish National Research Council (CSIC), Polytechnic University of Valencia, Valencia 46022, Spain

## Abstract

Sugars Will Eventually be Exported Transporters (SWEETs) have important roles in numerous physiological mechanisms where sugar efflux is critical, including phloem loading, nectar secretion, seed nutrient filling, among other less expected functions. They mediate low affinity and high capacity transport, and in angiosperms this family is composed by 20 paralogs on average. As SWEETs facilitate the efflux of sugars, they are highly susceptible to hijacking by pathogens, making them central players in plant–pathogen interaction. For instance, several species from the *Xanthomonas* genus are able to upregulate the transcription of SWEET transporters in rice (*Oryza sativa*), upon the secretion of transcription-activator-like effectors. Other pathogens, such as *Botrytis cinerea* or *Erysiphe necator*, are also capable of increasing *SWEET* expression. However, the opposite behavior has been observed in some cases, as overexpression of the tonoplast AtSWEET2 during *Pythium irregulare* infection restricted sugar availability to the pathogen, rendering plants more resistant. Therefore, a clear-cut role for SWEET transporters during plant–pathogen interactions has so far been difficult to define, as the metabolic signatures and their regulatory nodes, which decide the susceptibility or resistance responses, remain poorly understood. This fuels the still ongoing scientific question: what roles can SWEETs play during plant–pathogen interaction? Likewise, the roles of SWEET transporters in response to abiotic stresses are little understood. Here, in addition to their relevance in biotic stress, we also provide a small glimpse of SWEETs importance during plant abiotic stress, and briefly debate their importance in the particular case of grapevine (*Vitis vinifera*) due to its socioeconomic impact.

## Teaser

Plant SWEETs are sugar transporters important in plant development, plant–pathogen, and symbiotic interactions, but also in abiotic stress response and other plant cellular and physiological processes.

## Introduction

During most plant–pathogen interactions nutrients move from the plant to the microbe while the host cells try to restrict this transfer by reprogramming its carbon metabolism and transport (Chen et al., 2010; Kretschmer et al., 2017; Kanwar and Jha, 2019). During this clash, the photosynthetic activity is negatively affected and the related genes repressed (Chou et al., 2000; Lopes and Berger, 2001; Meyer et al., 2001; Berger et al., 2004, 2006; Zimmerli et al., 2004; Scholes and Rolfe, 2009; Chandran et al., 2010; Rolfe and Scholes, 2010; Windram et al., 2012; De Cremer et al., 2013; Smith et al., 2014), while genes of the respiratory process, e.g. glycolysis, tricarboxylic acid cycle, and mitochondrial electron transport chain are upregulated (Doehlemann et al., 2008; Parker et al., 2009; Chandran et al., 2010; Voll et al., 2011; Teixeira et al., 2014; Xu et al., 2015). The role of modulating sugar metabolism/transport in infection is exemplified in that soluble sugar content of maize (*Zea mays*) leaves is greatly altered during infection by the biotrophic pathogen *Ustilago maydis* (Doehlemann et al., 2008; Horst et al., 2008) and maize mutants with defects in sugar accumulation (*id1*: *indeterminate1*; increased accumulation of sucrose) or starch metabolism (*su1*: *sugary1*; altered starch metabolism) display reduced susceptibility to *U. maydis* infection (Kretschmer et al., 2017).

Modifications of the sugar metabolism during infection, including the activity of enzymes related with sugar hydrolysis and sugar transporters, favor the establishment of a sink-type environment in the infected tissue (Chou et al., 2000; Fotopoulos et al., 2003; Voegele et al., 2006; Hren et al., 2009; Hayes et al., 2010; Brzin et al., 2011; Cabello et al., 2014; Teixeira et al., 2014; Fatima and Senthil-Kumar, 2015; Dhandapani et al., 2016; Chang et al., 2017; Oliva and Quibod, 2017). In wheat (*Triticum aestivum*), the hexose transporter *Leaf Rust 67* (*Lr67*), also named *Sugar Transporter 13* (*STP13*), plays a key role in the susceptibility to all wheat rust and powdery mildew pathogen species. The dominant resistant variant *Lr67res* encodes a protein unable to transport sugars while the susceptible variant *Lr67sus* encodes a fully functional hexose transporter and both variants are upregulated when plants are challenged by pathogens. Thus, alterations in the hexose transport capacity, depending on the existing allele, may explain the ability of *Lr67res* to resist multiple pathogenic species (Moore et al., 2015). In Arabidopsis (*Arabidopsis thaliana*) leaves challenged with *Botrytis cinerea*, the expression of *STP13* is greatly increased. *stp13* mutant plants exhibit enhanced susceptibility and reduced rates of glucose uptake, while STP13 overexpressing plants show a resistant phenotype and higher glucose transport capacity involved in the active resorption of hexoses from the apoplast, depriving the pathogen from its sugar source (Lemonnier et al., 2014). Moreover, during bacterial attack, when the plant bacterial-flagellin receptor, Flagellin-sensitive 2 (FLS2) recognizes the bacterial flagellin peptide Flg22, the BRASSINOSTEROID INSENSITIVE 1-associated receptor kinase 1 (BAK1) phosphorylate STP13 increasing its sugar uptake capacity from the apoplast region, reducing the available sugar to the pathogen and increasing plant resistance (Yamada et al., 2016). In grapevine, *Hexose Transporter 5* (*VvHT5*) is strongly upregulated in coordination with *Cell Wall Invertase* (*VvcwINV*) during powdery (*Erysiphe necator*) and downy mildew (*Plasmopara viticola*) infection, which likely enhances sink strength during infection (Hayes et al., 2010). In a recent finding, grapevine’s sucrose transporter Early-Response to Dehydration six-like 13 (VvERD6l13) was also demonstrated to be upregulated in response to *E. necator* and *B. cinerea* infection (Breia et al., 2020a). In maize, expression of Sucrose Transporter 1 (*ZmSUT1*) is enhanced when challenged with the pathogen *Colletotrichum graminicola* (Vargas et al., 2012).

More recently a new type of sugar transporters, coined as SWEET (from Sugars Will Eventually be Exported Transporters), were identified in Arabidopsis by Chen et al. (2010) who tried to find the molecular basis that could explain sugar efflux mechanisms, which remained puzzling until then (Thorens et al., 2000; Stümpel et al., 2001; Hosokawa and Thorens, 2002; Lalonde et al., 2004). They screened genes encoding uncharacterized polytopic membrane proteins from the *Arabidopsis* membrane protein database Aramemnon (Schwacke et al., 2003) using a new mammalian expression system (Takanaga and Frommer, 2010). Candidate genes were co-expressed with the high-sensitivity förster resonance energy transfer (FRET) glucose sensor FLIPglu600mD13V in human HEK293T cells, with low endogenous glucose uptake activity (Takanaga et al., 2008; Takanaga and Frommer, 2010). AtSWEET1 (*AT1G21460*) was the first characterized SWEET transporter as a glucose bidirectional uniporter/facilitator (Chen et al., 2010). Also, to determine the bidirectional capacity of the transporter the FRET glucose sensor FLIPglu600mD13VER was expressed in the lumen of the endoplasmic reticulum. SWEET transporters have been classically characterized as uniporters that mediate both uptake and efflux of sugars in a low affinity and high capacity manner and relative pH independence, important in intracellular and intercellular sugar translocation. Also, they are strongly induced upon pathogen invasion (both bacteria and fungi), as nicely reviewed by Eom et al. (2015). However, since then, as discussed further ahead in this review, various new functional and physiological roles have been attributed to plant SWEET transporters, some more expected than others.

SWEET transporters belong to a transporter family (PFAM PF03083) whose members are highly conserved from the super kingdoms Archea and Bacteria (SemiSWEET family) to Fungi, Protista, and Metazoa. They are also present in Streptophyta (green plants), Chlorophyta (green algae), and other algae and even in the Oomycota class (Jia et al., 2017). This family is ubiquitously present in plants. As examples, in Arabidopsis it is constituted by 17 members (Chen et al., 2010), 21 in rice (Yuan and Wang, 2013), 23 in sorghum (*Sorghum bicolor*; Mizuno et al., 2016), 52 in soybean (*Glycine max*; Patil et al., 2015), 35 in potato (*Solanum tuberosum*; Manck-Götzenberger and Requena, 2016), 29 in tomato (*Solanum lycopersicum*; Feng et al., 2015), and 33 in apple (*Malus domestica*; Wei et al., 2014; Table 1).

**Table 1:** SWEET families of several plant species

Species	Number of SWEET members	Reference
*Arabidopsis thaliana*	17	Chen et al. (2010)
*Oryza sativa*	21	Yuan and Wang (2013)
*Vitis vinifera*	17	Chong et al. (2014)
*Manihot esculenta*	23	Cohn et al. (2014)
*Malus domestica*	33	Wei et al. (2014)
*Citrus sinensis*	16	Zheng et al. (2014)
*Amborella trichopoda*	8	Eom et al. (2015)
*Eucalyptus grandis*	47	Eom et al. (2015)
*Physcomitrella patens*	6	Eom et al. (2015)
*Solanum lycopersicum*	29	Feng et al. (2015)
*Glycine max*	52	Patil et al. (2015)
*Zea mays*	24	Sosso et al. (2015)
*Medicago truncatula*	26	Kryvoruchko et al. (2016)
*Solanum tuberosum*	35	Manck-Götzenberger and Requena (2016)
*Sorghum bicolor*	23	Mizuno et al. (2016)
*Gossypium hirsutum*	55	Cox et al. (2017)
*Cucumis sativus*	17	Hu et al. (2017)
*Pyrus bretschneideri*	18	Li et al. (2017a)
*Musa acuminata*	25	Miao et al. (2017)
*Lotus japonicus*	13	SugiyaMa et al. (2017)
*Hevea brasiliensis*	36	Sui et al. (2017)
*Triticum aestivum*	59/108	Gao et al. (2018), Gautam et al. (2019)
*Ananas comosus*	39	Guo et al. (2018)
*Saccharum spontaneum*	22	Hu et al. (2018)
*Brassica rapa*	32	Li et al. (2018a)
*Camellia sinensis*	13	Wang et al. (2018a)
*Pisum sativum*	26	Doidy et al. (2019)
*Fragaria vesca*	20	Liu et al. (2019)
*Litchi chinensis*	16	Xie et al. (2019)
*Brassica oleracea*	30	Zhang et al. (2019a)
*Ziziphus jujuba*	19	Geng et al. (2020)
*Juglans regia*	25	Jiang et al. (2020)
*Populus trichocarpa*	27	Zhang et al. (2020a)
*Poa pratensis*	13	Zhang et al. (2020b)

These transporters are structurally different from the classic 12 transmembrane-domains sugar transporters previously characterized of the major facilitator superfamily. They are composed by two internal triple-helix bundles linked by a linker-inversion transmembrane domain (TMD), comprising seven TMDs in total (Chen et al., 2010). Bacterial SemiSWEET are formed by only three TMDs and structural resolution studies showed that two individual SemiSWEET transporters form oligomers in parallel orientation to create a functional pore for translocation. Therefore, SWEETs possibly arose by gene duplication of SemiSWEET units in concert with the insertion of an inversion linker-helix (Xuan et al., 2013; Xu et al., 2014; Wang et al., 2014). More recently, following an extensive phylogenetic analysis, Hu et al. (2016) proposed that a fusion of archeal and bacterial SemiSWEETs formed eukaryotic SWEETs, which potentially explains the asymmetry of eukaryotic SWEETs. Still, how the least conserved TMD4 was inserted in the structure remains unclear.

Although crystal structures and molecular dynamic simulations were published, the detailed mechanism of this family of sugar transporters is being unveiled. The structure and regulation of SWEET transporters in plants has recently been reviewed (Anjali et al., 2020) so this topic is out of the scope of the present paper. In Arabidopsis SWEET proteins, four conserved prolines have a significant role in the transport mechanism. In AtSWEET1, replacing any of the four prolines caused loss of AtSWEET1 activity (Tao et al., 2015). SWEET transporters can also form oligomers, as structural and biochemical analyses showed that OsSWEET2b forms homomeric trimers (Tao et al., 2015). The crystal structure of AtSWEET13, a multisubstrate transporter, was also reported as in an inward facing conformation with a sucrose analog bound in the central cavity (Han et al., 2017). It was shown that in response to substrate binding, different parts of the cytosolic side of AtSWEET13 move independently, instead of forming rigid bodies. A revolving-door like mechanism for transport by an AtSWEET13 dimer was postulated, in which a substrate-carrying conformational transition in one protomer is coupled to the substrate-free opposite transition in the other protomer. Additionally, SWEETs contain multiple phosphorylation sites at the cytosolic C-terminal end with an average of approximately 45 amino acids. The cytosolic C-terminus may act as a hub for binding of other proteins (e.g. regulatory components), or it could function in transmission of signals to the cell if SWEETs also function as sugar receptors (or transceptors; Chen et al., 2015b).

## SWEET roles in plant growth and development: more than just sugar loading and unloading

Plant SWEET sugar transporters play different physiological roles during plant growth and development. In angiosperms, this family is constituted on average by 20 paralogs (Table 1), differentially expressed in several tissues. SWEET members are phylogenetically divided in four clades; however, membership in a clade does not predict the physiological role of the protein, but slightly defines its substrate specificity. In Arabidopsis, clade I (SWEET1-2), II (SWEET3-8), and IV (SWEET16-17) predominantly transport monosaccharides while clade III (SWEET9-15) mediates mainly sucrose uptake (Chen et al., 2015b). Likewise, SWEET transporters can localize in different cellular compartments, mainly in the plasma membrane (SWEET1, 8, 9, 11, 12, and 15; Seo et al., 2011; Kryvoruchko et al., 2016), but also in the tonoplast (SWEET2, 16, and 17; Chardon et al., 2013; Klemens et al., 2013; Guo et al., 2014; Chen et al., 2015a) and in the Golgi membrane (SWEET9; Lin et al., 2014; Chen et al., 2015b).

**Figure 1:**
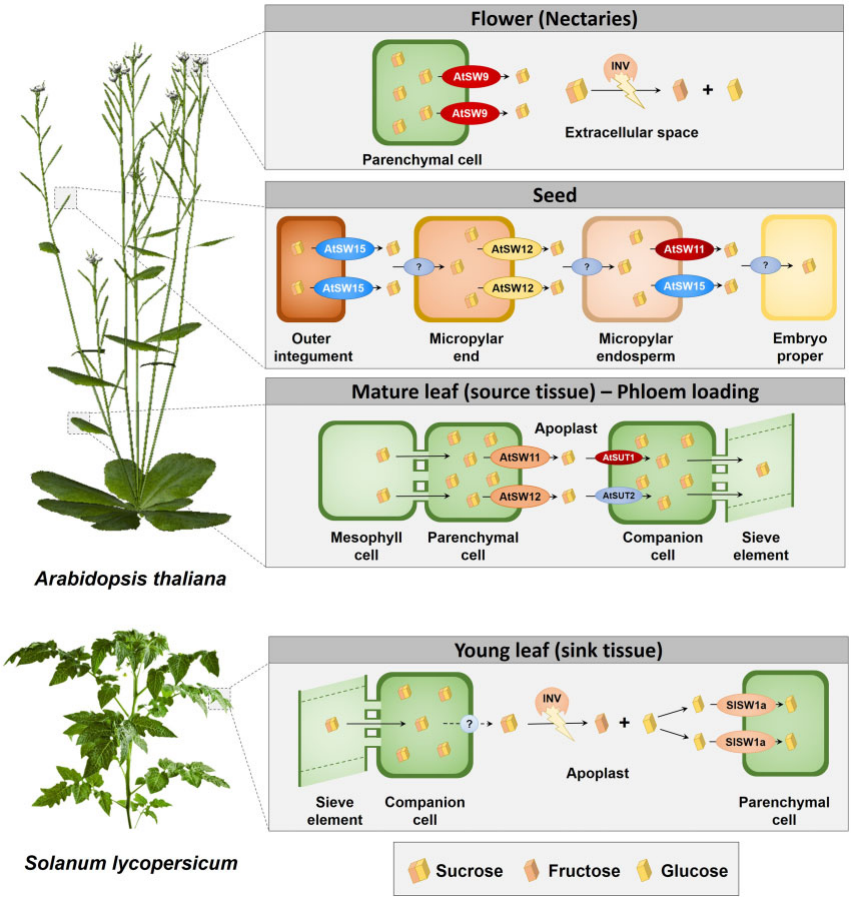
Diverse physiological roles of SWEET transporters in different organs across different plant species. Plant SWEET transporters are pivotal during phloem loading, transporting sucrose from the parenchyma cells to the apoplast by a facilitated diffusion (AtSWEET11 and 12). Sucrose is then loaded to the phloem by symporters (Chen et al., 2012). In flower nectaries, sucrose efflux is mediated by SWEET transporters (AtSWEET9—Lin et al., 2014). SWEET transporters (SlSWEET1a) also participate in the uptake of glucose from the apoplast to the parenchyma cells of young tomato leaves (Ho et al., 2019) and during seed filling (AtSWEET11, 12, and 15) in a sequential manner (Chen et al., 2015c). SW, SWEET; INV, Invertase; AtSUT1, *Arabidopsis thaliana* Sucrose Transporter 1; AtSUT2, *Arabidopsis thaliana* Sucrose Transporter 2.

Sucrose synthesized in the leaf mesophyll is transported to the apoplast by facilitated diffusion and then actively incorporated by plasma membrane sucrose/proton symporters (SUT/SUC) in the companion cells or sieve element cells (Figure 1; Lalonde et al., 2004; Sauer, 2007; Kühn and Grof, 2010; Slewinski et al. 2010; Ainsworth and Bush, 2011; Ayre, 2011). The molecular mechanisms involved in the export of sucrose to the apoplast remained unsolved until the discovery that SWEET transporters are key elements during phloem loading by Chen et al. (2012). Sucrose transporters *AtSWEET11* and *12* were found highly expressed in a subset of leaf phloem parenchyma cells, proximal to the companion cells and sieve elements. Double mutant *atSWEET11;12* lines showed moderate defects in sucrose phloem transport and an excessive accumulation of sugars in the leaves and delayed root development. Remarkably, AtSWEET11 and 12 are also present in Arabidopsis xylem vessels and as the double mutant *atSWEET11;12* showed severe modifications in the chemical composition of the xylem cell walls it was suggested that these transporters export carbon skeletons to developing xylem cells in order to support secondary cell wall formation (Le Hir et al., 2015). The rice homolog *OsSWEET11* is expressed in the phloem of rice leaves (Chu et al., 2006), indicating that it may play a similar role in phloem loading as *AtSWEET11* and *12*, and in *Ziziphus jujuba*, overexpression of *ZjSWEET2.2* increased carbon fixation to some extent in photosynthetic organs, suggesting that it stimulates SWEET-mediated phloem loading of photoassimilates (Geng et al., 2020). In sorghum, *SbSWEET13a*, *13b*, and *13c* are mostly expressed in leaves and stems and their expression pattern corresponds with sucrose accumulation in the stem (Makita et al., 2015; Bihmidine et al., 2016). In maize, similarly to *ZmSUT1*, *ZmSWEET13a*, *13b*, and *13c* are preferentially expressed in the bundle sheath/vein of leaves. Triple knockout mutants of *ZmSWEET13a, b, c* showed a severely stunted phenotype, with impaired phloem loading, reduced photosynthetic activity, and accumulation of high levels of soluble sugars and starch in leaves. Furthermore, RNA-seq analysis revealed a deep transcriptional deregulation of genes associated with photosynthesis and carbohydrate metabolism (Bezrutczyk et al., 2018a). In potato, StSWEET11 is a plasma membrane sucrose transporter pivotal for phloem loading. *StSWEET11 RNAi* lines showed a reduction in yield and accumulate starch and sucrose in leaves (Abelenda et al., 2019).

SWEET transporters also play crucial roles in sugar unloading from phloem complexes to sink tissues through the apoplastic pathway (Figure 1). In young tomato leaves (sinks), *SlSWEET1a*, a glucose transporter, is strongly expressed in the unloading veins tissues, as observed by GUS staining. Moreover, in mutant tomato plants with suppressed expression of *SlSWEET1a* by virus-induced gene silencing, the concentration of glucose and fructose is significantly reduced in young leaves but increases in mature leaves. Altogether, these results support a crucial role of SlSWEET1a in the uptake of glucose from the apoplast to the parenchyma cells in sink tissues, maintaining a lower turgor pressure toward phloem cells for continuous sugar import to these tissues (Ho et al., 2019). In sink tissues of potato like the stolon and tubers, StSWEET11 is expressed in the phloem companion cells and participates in the leakage of sucrose in the apoplast. Thus, apoplastic sugar content in stems was higher in *35S:StSWEET11* plants and lower in *StSWEET11 RNAi* ones. During tuber formation, the FLOWERING LOCUS T-like protein of potato, StSP6A, which is essential for tuberization (Navarro et al., 2011), is expressed in the phloem of apical and subapical meristem of the stolon and interacts with StSWEET11, blocking sucrose leakage to the apoplast. This blockage switches the sucrose unloading in the tuber from apoplastic to symplastic, indicative of tuber formation (Viola et al., 2001). These evidences show that the cross-talk between SWEET transporters and other proteins is finely tuned to regulate sink–source relationships (Abelenda et al., 2019).

In Arabidopsis, AtSWEET4 is expressed in the stele of roots and veins of leaves and flowers. Overexpression mutant lines showed increase plant size and more glucose and fructose, while knockdown mutant lines were smaller, contained lower amounts of glucose and fructose, and less chlorophyll in leaves, suggesting that AtSWEET4 mediates sugar transport in axial tissues (Liu et al., 2016). During senescence of Arabidopsis leaves, the sucrose transporter *AtSWEET15* is strongly upregulated (Quirino et al., 1999) and *AtSWEET15*-overexpressing lines exhibited enhanced leaf senescence, suggesting a function of AtSWEET15 in sugar remobilization (Seo et al., 2011). AtSWEET15 homolog in pear (*Pyrus bretschneideri*), PbSWEET4 is also implicated in leave senescence. Heterologous overexpression of *PbSWEET4* in strawberry plants caused reduced leaf sugar and chlorophyll content and accelerated leaf senescence (Ni et al., 2020).

SWEET transporters are also key players in plant reproductive organs, such as flowers, fruits, and seeds, where the efflux of sugar from the autotrophic tissues is of utmost importance. Concordantly, most of the reported SWEET transporters from different species have been associated with these plant tissues. Transcriptomic experiments in rice showed that 17 *SWEET* genes are highly expressed in flowers and seeds (Yuan et al., 2014). In cucumber (*Cucumis sativus*), most *SWEET* genes are confined to reproductive tissue development (Hu et al., 2017) and similar results were observed in the Chinese white pear (Li et al., 2017a). In pineapple (*Ananas comosus*), different *SWEET* genes are strongly expressed during fruit development, of which *AnmSWEET5* and *AnmSWEET11* display the highest transcript abundance (Guo et al., 2018). Likewise, from a total of 25 *MdSWEET* genes identified in apple genome, 9 are highly expressed during fruit development. Among them, *MdSWEET2e*, *MdSWEET9b*, and *MdSWEET15a* were linked with fruit sugar accumulation, with *MdSWEET9b* and *MdSWEET15a* as the main contributors for the major proportion of phenotypic variation in sugar concentration among different cultivars (Zhen et al., 2018). In loquat (*Eriobotrya japonica*), higher expression of *EjSWEET15* is linked with higher sugar concentration cultivars (Li et al., 2020) and in cotton (*Gossypium hirsutum*), transcriptional data and promoter analyses in *SWEET* genes point to the involvement of these transporters in cotton fruit development processes (Li et al., 2018b), with the same approach indicating a similar role in litchi (*Litchi chinensis*; Xie et al., 2019).

In Arabidopsis, AtSWEET8 is involved in the transport of glucose for pollen nutrition. This transporter is highly expressed in the tapetum and *atSWEET8* mutant lines shows male sterility, which results in nonviable pollen grains (Guan et al., 2008). Likewise, *AtSWEET15* (also known as *Vegetative Cell Expressed 1*, *VEX1*) is highly expressed in pollen grains and involved in the transport of sugars, especially in the vegetative cells. This transporter is also continuously expressed during pollen maturation and even in germinating pollen grains (Engel et al., 2005), indicating an important physiological role of this transporter during pollen development. In rice, the sucrose transporter OsSWEET11, which is highly expressed in pollen grains, has a prominent role in pollen viability as pollen grains of *OsSWEET11* knockout mutants showed reduced starch contents, which may lead to male sterility (Yang et al., 2006). Reinforcing the importance of some SWEETs in plant reproduction, in *Jasminum sambac*, seven SWEET transporters are sequentially expressed during flower development (Wang et al., 2019a) and in Arabidopsis, eight SWEET genes are highly expressed (*AtSWEET15*, *14*, *13*, *8*, *7*, *5*, *4*, and *1*). *AtSWEET14* and *13* predominate in the stamen; *AtSWEET8* is abundant in the microspores; and *AtSWEET15* and *1* are abundant in the petals and *AtSWEET4* in the sepals (Moriyama et al., 2006). *AtSWEET10* is transcriptionally activated by FLOWERING LOCUS T-signaling pathway during floral transition under a photoperiod-dependent manner. Overexpression of AtSWEET10 causes early flowering, which suggest the importance of sugar transport during floral transition (Andrés et al., 2020).

SWEET transporters play also pivotal roles in nectar secretion (Figure 1). AtSWEET9 in Arabidopsis, *Brassica rapa*, and *Nicotiana attenuata* (all eudicots) mediates sugar efflux in the nectary parenchyma. Loss-of-function mutants lead to loss of nectar secretion in all the studied plants. The secretion of sucrose into the extracellular space is then hydrolyzed into glucose and fructose which maintains the concentration gradient (Lin et al., 2014). AtSWEET9 homolog in *Petunia hybrida*, NEC1, is also nectary-specific and its expression pattern corresponds inversely with nectarial starch content. Likewise, silencing this gene triggered male sterility (Ge et al., 2000, 2001).

Arabidopsis *SWEET15*, *11*, and *12* are highly expressed in the seed coat suggesting a key role in seed development (Figure 1). Triple-knockout mutants showed a severe delay in embryo development and a wrinkled seed phenotype at maturity due to lower starch and lipid content and a smaller embryo. Thus, these proteins are involved in the transport of sucrose from the seed coat to the embryo in a coordinated manner (Chen et al., 2015c). In soybean, the sucrose transporters GmSWEET15a and 15b are highly expressed in the endosperm during the first phases of seed development. *GmSWEET15a;b* knockout mutants showed reduced embryo sugar content, retarded embryo development, and endosperm persistence, resulting in severe seed abortion. GmSWEET15a and 15b are then crucial for feeding sugars from the endosperm to the developing embryo (Wang et al., 2019b). Also, in soybean, the mono- and disaccharides transporters *GmSWEET10a* and *10b* are specifically expressed in the seed coat and contribute to sugar transport from seed coat to embryo. Resequencing data from over 800 genotypes revealed that selection of optimal *GmSWEET10a* and *10b* alleles are directly related with the increased soybean seed size, oil, and protein content of modern soybean varieties (Wang et al., 2020). Similarly, maize *SWEET4c* shows indicative signatures of selection during domestication. This hexose transporter is expressed in the basal endosperm transfer layer region and is responsible for transferring hexoses to sustain development of endosperm. Notably, *zmSWEET4c* insertion mutants showed grain defects, including a dramatic loss of endosperm. The rice homolog OsSWEET4 appears to have similar functions (Sosso et al., 2015). Two others SWEET transporters are essential for rice grain filling. During the early stages of caryopsis development, OsSWEET11 is particularly abundant in the nucellar epidermis, ovular vascular trace, and cross cells, playing an important role in sucrose release from maternal tissue to the maternal–filial interface. Knockout mutants of *OsSWEET11* showed significant reduction in sucrose concentration in the embryo sac, leading to defective grain filling, reduced grain weight, and seed setting percentage (Ma et al., 2017). OsSWEET15 is also crucial for seed filling. In fact, at latter seed developmental stages, OsSWEET11 and 15 are necessary for sugar efflux from the maternal nuclear epidermis as well as efflux from the ovular vascular trace to the apoplast, and also may contribute to sucrose influx into the aleurone. Double mutant plants exhibited accumulated starch in the seed pericarp, whereas caryopses did not contain a functional endosperm (Yang et al., 2018).

Vacuolar sugar transport and storage are tightly related with resistance to different environmental constraints (Martinoia et al., 2007). Likewise, *AtSWEET16* expression, a multisubstrate vacuolar transporter, is repressed under low nitrogen. Overexpressing lines (*35S_Pro_:SWEET16*) showed a number of peculiarities related to differences in sugar accumulation. During nitrogen starvation mutant lines accumulated glucose and fructose, but no sucrose. Remarkably, *35S_Pro_:SWEET16* lines showed improved germination and improved nitrogen use efficiency (Klemens et al., 2013). The Arabidopsis AtSWEET2 is highly expressed in the tonoplast of the root caps and tips and likely limits the carbon efflux from roots into the rhizosphere by accumulating sugars in the vacuole. Concordantly, *atSWEET2* mutants showed increased loss of glucose from the roots into the rhizosphere (Chen et al., 2015a).

Surprisingly, SWEET proteins can also be involved in hormone regulation. In Arabidopsis, AtSWEET13 and 14 can transport different gibberellins (GAs) and *atSWEET13;14* double-mutant lines were incapable of transporting exogenous GA and showed altered responses during seed germination (Kanno et al., 2016). In rice, OsSWEET3a is expressed in the vascular tissue of basal parts of seedlings and, besides glucose, it transports GAs. Knockout and overexpression mutant lines showed defects in germination and early shoot development, suggesting an involvement of OsSWEET3a in the transport of GA and glucose to young leaves during early plant development. Interestingly, it is suggested that GA transport activity of SWEETs evolved independently during plant evolution as in Arabidopsis it evolved from sucrose-specific SWEETs while in rice from glucose-specific ones (Morii et al., 2020). OsSWEET5 is a galactose transporter mainly expressed in the floral organs at the heading stage but also in stem, root, and senescing leaves. *OsSWEET5*-overexpressing plants showed growth retardation, precocious senescing leaves, and changed sugar contents in leaves. Remarkably, auxin concentration, signaling, and translocation were inhibited. *OsSWEET5* is possibly an important player in the sugar and auxin crosstalk (Zhou et al., 2014).

## SWEET roles in plant–pathogen and symbiotic interactions

**Figure 2:**
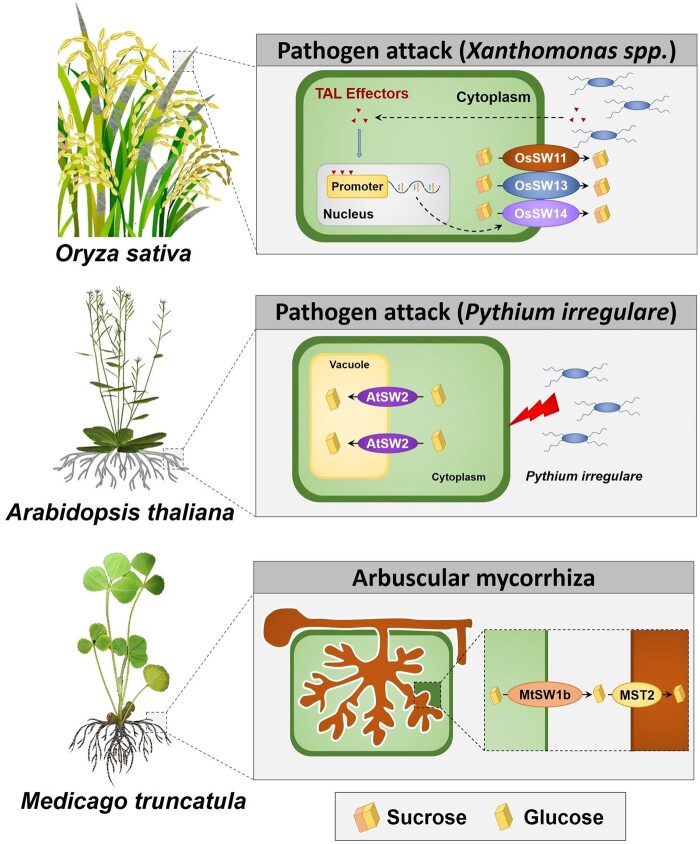
Involvement of SWEET transporters during plant–pathogen and symbiotic interactions. *Xanthomonas* spp. secretes TAL effectors that induce the expression of SWEET (e.g. OsSWEET11, 13, and 14) to increase sugar leakage (Chen et al., 2010). SWEETs can be also involved in defense mechanisms like in infected Arabidopsis roots in which AtSWEET2 increases the accumulation of cytosolic sugars in the vacuole, impeding its transport to the extracellular space (Chen et al., 2015a). During interactions with beneficial microorganisms, such as in *M. truncatula* with the symbiotic AM (*R. irregularis*), MtSWEET1b transports glucose across the peri-arbuscular membrane (An et al., 2019). SW, SWEET; MST2, AM fungus Monosaccharide Transporter 2.

Different SWEET transporters are upregulated in plants upon infection by different species of the genus *Xanthomonas* that cause bacterial blight disease (Yang et al., 2006; Antony et al., 2010; Chen et al., 2010; Liu et al., 2011; Yu et al., 2011). These bacteria secrete several transcription-activator like (TAL) effectors (Bogdanove, 2014) to directly enhance the expression of specific *SWEET* genes (Figure 2). Thus, *Xanthomonas oryzae* pv. *oryzae* secretes the TAL effector PthXO1 that targets rice *OsSWEET11* (Yang et al., 2006; Chen et al., 2010), and an African *Xanthomonas* strain secretes the effector TALC that increases the transcript abundance of *OsSWEET14* (Yu et al., 2011). *OsSWEET13* is also upregulated during *X. oryzae* pv. *oryzae* infection; however, no effector was identified (Liu et al., 2011). SWEET transporter activity hijacked by *Xanthomonas* species appears to be crucial for the growth and proliferation of the pathogens because the lack of induction results in disease resistance. Bacterial mutant strains carrying truncated versions of TAL effectors or even plant mutations in the promoter region where TAL effectors bind result in reduced bacterial titer (Chen et al., 2010; Liu et al., 2011; Yu et al., 2011). For instance, the strain PXO99A mutated in its *pthxo1* gene cannot induce *OsSWEET11* and fails to infect rice plants (Chen et al., 2010). Interestingly, the same SWEET member can be targeted by different pathogen strains specific effectors, as these effectors can bind to different regions of the same SWEET gene promoter. Thus, recessive mutations in the promoter region of *SWEET* genes can increase pathogen resistance without losing the sugar transport function (Antony et al., 2010; Yu et al., 2011). It seems that so far these pathogens target clade III SWEET transporters, with a physiological function normally related with sucrose efflux to the apoplastic space surrounding the phloem, including AtSWEET11 and AtSWEET12 (Chen et al., 2012). These bacterial pathogens also target SWEET transporters in other plant species. In Cassava (*Manihot esculenta*), *MeSWEET10a* is induced by *Xanthomonas axonopodis*, promoting its virulence (Cohn et al., 2014), and in citrus, the pathogen *Xanthomonas citri* ssp. *citri*, which causes bacterial canker disease, induces *CsSWEET1* by a TAL effector-dependent manner (Hu et al., 2014). Also, in cotton, the bacterial blight disease casual-agent, *X. citri* subsp. *malvacearum*, specifically activates GhSWEET10d, a sucrose transporter, by its effector Avrb6 (Cox et al., 2017).

It has been widely shown that *SWEET* gene expression can be altered not only by *Xanthomonas* species but also by other bacterial and fungal pathogens. *Pseudomonas syringae* may induce different *SWEET* genes (*AtSWEET4*, *5*, *7*, *8*, *10*, *12*, and *15*) in infected Arabidopsis leaves, especially *AtSWEET15* during the early stages of infection (Chen et al., 2010). Also, in Arabidopsis, infection by the obligate biotrophic pathogen *Golovinomyces cichoracearum* induced the expression of *AtSWEET12* in infected leaves during the formation of the primary haustorium and during hyphal growth and development of the reproductive structures (Chen et al., 2010). Infection by *Rhizoctonia solani*, the causative agent of sheath blight disease, which is a major pathogen of rice, significantly induced *OsSWEET11* expression in leaves. The analyses of transgenic plants revealed that *OsSWEET11* mutants were less susceptible, whereas plants overexpressing *OsSWEET11* were more susceptible to sheath blight compared with wild-type controls (Gao et al., 2018). *Botrytis cinerea* infection also enhanced the expression of different *AtSWEETs*, principally *AtSWEET15* (Chen et al., 2010). *Botrytis cinerea* also induces *SlSWEET15* in tomato (Asai et al., 2016).

Overall, it seems that most of the pathogens induce host SWEET transporters to gain access to the plant sugar resources for nourishment (Chen et al., 2010; Cohn et al., 2014) as host-derived sugars are unequivocally absorbed by the pathogen (Aked and Hall, 1993; Sutton et al., 1999). However, in certain interactions, it is not clear if the increased expression of SWEET transporters is mediated by the pathogen or is itself a host response to prevent pathogen infection. In fact, in some cases, upregulation of these transporters does not result in higher plant susceptibility to infection (Figure 2). In roots of Arabidopsis, despite infection by the soil-borne oomycete *P. irregulare* caused an increase of more than 10-fold in *AtSWEET2* gene expression, the loss-of-function *SWEET2* mutants were more susceptible to the infection, showing impaired growth when challenged with the oomycete and suggesting that AtSWEET2 transporters retrieve sugars from the cytosol to the vacuole to limit their leakage to the extracellular space where they may feed the pathogen (Chen et al., 2015a). In sweet potato (*Ipomoea batatas*), infection with *Fusarium oxysporum* Schlecht. f. sp. *batatas* significantly upregulated the gene expression of the sucrose transporter *IbSWEET10*. Unexpectedly, *IbSWEET10*-overexpressing sweet potato lines were more resistant against this fungal pathogen than control ones and also RNAi lines showed higher susceptibility (Li et al., 2017b). The mechanisms by which higher levels of SWEET activity increase plant resistance to pathogenic attack are still poorly understood. One hypothesis is that SWEET transport activity lowers the available sugar in the apoplast thus affecting fungal growth. Also, as sugars can act as signaling molecules, SWEET upregulation may alter sugar levels at the infection site and trigger signaling cascades that result in the salicylic acid or jasmonic acid pathways activation, and consequently the upregulation of defense genes (Herbers et al., 1996; Herbers and Sonnewald, 1998; Morkunas and Ratajczak, 2014; Gebauer et al., 2017; Bezrutczyk et al., 2018b; Kanwar and Jha, 2019). Still, SWEETs can possibly function as sugar sensors much like other sugar transporters, including SUC2 and SUT1 in Arabidopsis (Lalonde et al., 1999; Barker et al., 2000; Thevelein and Voordeckers, 2009), or Sucrose Nonfermenting 3 (SNF3) and Restores Glucose Transport 2 (RGT2) in *Saccharomyces cerevisiae* (Özcan et al., 1998); however, this hypothesis is still highly speculative (Bezrutczyk et al., 2018b).

Contrarily to the above reported data, downregulation of several *SWEET* genes in tomato cotyledons was observed when challenged with *B. cinerea* (Asai et al., 2016). Over 21 of the 30 *SlSWEET* genes were significantly downregulated 16 h after inoculation. The physiological importance of downregulation of *SWEET* genes during infection is still puzzling. It was reported that upon pathogen attack various sugar signaling cascades are disrupted (Berger et al., 2006; Sade et al., 2013; Morkunas and Ratajczak, 2014) eventually due to the downregulation of *SWEET* genes. Therefore, pathogens could repress these transporters to decrease plant defense responses resulting in a more beneficial environment for pathogen growth.

SWEET family members are also induced upon plant interaction with mycorrhizal fungi and rhizobia bacteria (Perotto et al., 2014; Kryvoruchko et al., 2016). During the symbiotic nitrogen fixation process that occurs in *Medicago truncatula* root nodules, the sucrose transporter *MtSWEET11* is highly expressed. This transporter is present in the root hair cells, in the meristem, invasion zone, and vasculature of nodules. However, MtSWEET11 is not crucial for symbiotic nitrogen fixation as in mutant *MtSWEET11* lines this symbiosis was uncompromised (Kryvoruchko et al., 2016). In *Lotus japonicus*, 13 members of the SWEET family were expressed in nodules. During nodule development, *LjSWEET3* was highly expressed, reaching the highest level in mature nodules, suggesting a sugar translocation function toward nodules (SugiyaMa et al., 2017). In potato, the arbuscular mycorrhizal (AM) fungus *Rhizophagus irregularis* greatly modified the expression profile of 22 *SWEET* genes, upregulating 12 members and repressing 10 (Manck-Götzenberger and Requena, 2016). In *M. truncatula*, *SWEET1b* transporter is strongly upregulated in arbuscule containing cells compared with roots and localizes to the peri-arbuscular membrane in the cortical cells, across which nutrient exchange takes place (Figure 2). Overexpression of *MtSWEET1b* in *M. truncatula* roots promoted the growth of intraradical mycelium during AM symbiosis, increasing the fungal mass and the overexpression of *MtSWEET1b^Y57A/G58D^*, which are considered to act in a dominant-negative manner, resulted in enhanced collapse of arbuscules. Therefore, these results suggested that MtSWEET1b is strictly related with sugar transport to AM fungi, however, in a redundant manner (An et al., 2019).

## The particular case of grapevine SWEET transporters during pathogen attack

Grapevine is a plant species highly susceptible to different diseases, including gray mold (*B. cinerea*), powdery mildew (*E. necator*), and downy mildew (*P. viticola*), that seriously threaten grape growers. In grapevine, *B. cinerea* can infect several tissues at different developmental stages and negatively affects grape berry production and quality (Gomès and Coutos-Thévenot, 2009; Walker and Leroux, 2015). Nevertheless, it can also cause noble rot, a disease that only develops under very specific edaphoclimatic conditions, leading to the production of exceptionally SWEET and high-quality wines (Magyar, 2011; Vannini and Chilosi, 2013; Jackson, 2020). Obtaining and taking advantage of the perfect conditions for noble rot development is currently a significant challenge, but one that may provide large rewards in the future. In this regard, full knowledge on SWEETs functional and physiological roles in grapevine, particularly in grape berries, is of utmost importance, as it would greatly contribute to understand and find the right “balance” between infection and plant health and thus the development of optimal *B. cinerea*-infected berry characteristics, providing in a near future the much needed standardization and increase in the production of these added-value SWEET/fortified wines. Moreover, the consumer demand for these exceptional and unique wines is increasing (Jackson, 2020), so, to understand SWEET involvement in this context, and even to tame and exploit their role and potential is currently a market-driven priority.

The role of sugar transporters in grapevine/environment interaction has been widely reported (Hayes et al., 2010; Santi et al., 2013; Conde et al., 2015; Cai et al., 2019; Breia et al., 2020b); however, Chong et al. (2014) showed for the first time that a grapevine SWEET transporter (VvSWEET) is involved in a pathogen attack microenvironment, right after the in silico identification of the VvSWEET family by Lecourieux et al. (2014). This family is composed by 17 members named based on their sequence identity percentage with Arabidopsis SWEET proteins which comprise a family with the same number of members. A previous study proposed *VvSWEET17b* and *VvSWEET17c* as a single gene with a 14-TMD extraSWEET (Patil et al., 2015), but this assumption resulted from an error in the 12× Genoscope annotation, which is not present in the new grapevine genome annotation (VCost.v3; Canaguier et al., 2017). VvSWEETs clearly separate in the classic four clades; however, clade III appears to be underrepresented. *VvSWEET* genes are differentially expressed in each grapevine organ and only *VvSWEET9* and *17b* expression was not detected so far. Numerous members are highly expressed in reproductive organs and fewer in vegetative ones. Both VvSWEET4 (Chong et al., 2014) and VvSWEET10 (Zhang et al., 2019b) were functionally characterized as plasma membrane glucose transporters. The overexpression VvSWEET10 in grapevine *calli* and tomato increased the glucose, fructose, and total sugar levels, suggesting that this transporter is an important player during sugar accumulation in grape berry (Zhang et al., 2019b).

**Figure 3:**
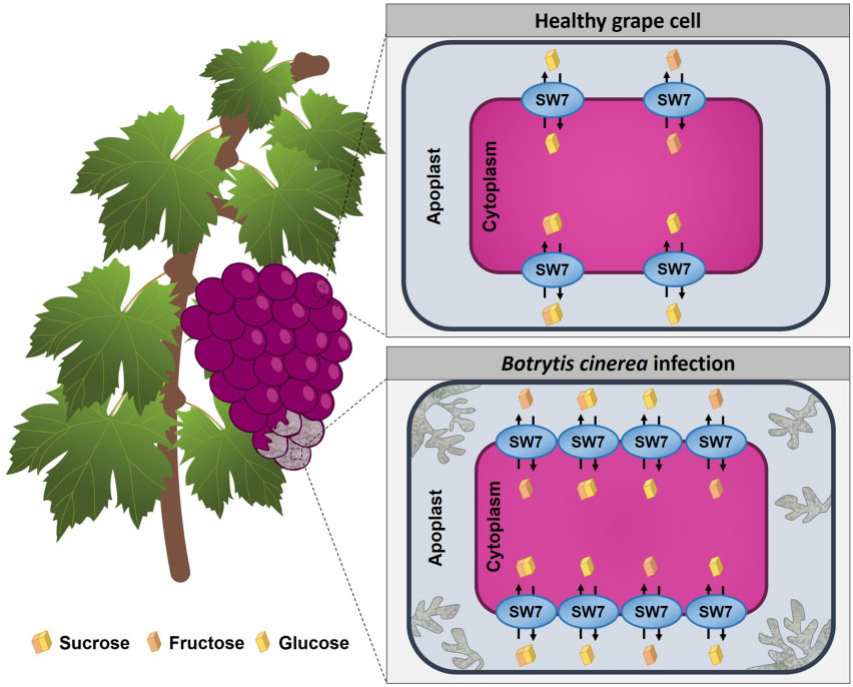
VvSWEET7 during *Botrytis cinerea* grape berry infection. The mono- and disaccharide transporter VvSWEET7 is strongly upregulated during *B. cinerea* infection of grape berries (Breia et al., 2020b). This induction may be caused by the pathogen itself to promote leakage of sugars into the apoplastic space for nutrition, or, as a defense-related process to improve sugar remobilization which can trigger signaling cascades that activate plant-defense mechanisms. SW, SWEET.

Recently, two grapevine SWEET members (VvSWEET7 and 15) were studied in our group, and both proteins were found highly expressed during grape berry development, at the green and mature stages (Figure 3; Breia et al., 2020b). Concordantly, the expression levels of *VvSWEET15* is positively associated with hexose contents in different varieties of grape berries (Ren et al., 2020). Both VvSWEET7 and 15 are localized in the plasma membrane, and the heterologous expression in yeast of VvSWEET7showed that it mediates a high-capacity, low-affinity transport of mono and disaccharides, but interestingly it also permeates substrates like polyols (Breia et al., 2020b). In field-trials, *B. cinerea* infection of Trincadeira cv grape berries caused a strong reprogramming of the expression of several *VvSWEET* genes. *VvSWEET7* and *15* were clearly upregulated in response to infection, as well as *VvSWEET2a*. But *B. cinerea* infection also downregulated *VvSWEET10*, *11*, *17a*, and *17d* expression at different developmental stages (Breia et al., 2020b). The observation that VvSWEET7 also transport polyols is particularly relevant because many pathogens synthetize the sugar-alcohol mannitol as a mechanism for neutralizing the oxidative burst of plants in response to the infection (Patel and Williamson, 2016). Thus, VvSWEET7 may be involved in plant defense by readily removing from the apoplast the pathogen-synthetized mannitol.

Much like in other plant species, grapevine *VvSWEETs* are transcriptionally reprogrammed during infection. The grapevine biotrophic pathogens *E. necator* and *P. viticola* did not significantly induce *VvSWEET* expression, while the infection with the necrotroph *B. cinerea* triggered a strong upregulation of *VvSWEET4* expression (Chong et al., 2014). This sugar transporter is also induced by ROS production, cell death and virulence factors from necrotizing pathogens, all hallmarks of necrotrophic interactions. The overexpression of *VvSWEET4* in grapevine hairy roots improved the resistance to *P. irregulare* infection. In parallel, glucose concentration increased and the upregulation of different genes of the flavonoid biosynthesis accounted for the observed higher flavanol contents. Altogether, these results suggest an involvement of VvSWEET4 in biotic defense mechanisms in grapevine (Meteier et al., 2019).

In sum, *VvSWEET* transporters are likely important players in sugar mobilization during grape berry development and their expression is transcriptionally reprogrammed in response to *B. cinerea* infection. However, the role of SWEETs in plants as components of susceptibility or resistance during pathogenic attack is still a matter of debate and may only be resolved by a case by case basis. Besides its scientific relevance, the knowledge on grapevine SWEET transporters in plant–pathogen interaction may provide cues for the optimization of agricultural practices toward increased vine health, grape berry, and wine productivity. Also, it may open or widen new avenues for the optimization of new and more unique wines as the case of the recently trendy and expensive “Botrytized wines” (Magyar and Soós, 2016; Jackson, 2020).

## SWEETs transporters during abiotic stress—a small glimpse on a broader function of SWEETs in plant–environment interactions?

Evidence of the role of SWEET transporters during abiotic stresses is still scarce and fragmented but different reports suggest their involvement in plant response to cold, high temperature, drought, and salinity. As shown below, these conditions may require the intracellular accumulation of compatible solutes/osmolytes, putatively mediated by SWEETs, to protect cell proteins against dehydration/denaturation (Ruan et al., 2010; Wang et al., 2018b). Yet, because limited water availability drastically reduces photosynthesis and plant carbon assimilation (Moutinho-Pereira et al., 2004; Chaves et al., 2009), it is somewhat expected that drought stress may indirectly modify the expression of different sugar transporters to maintain cellular homeostasis (Yamada et al., 2010, 2011; Schulz et al., 2011; Frost et al., 2012; Osakabe et al., 2014; Gong et al., 2015).

In Arabidopsis, banana (*Musa acuminata*), rice, tea plant (*Camellia sinensis*), *M. truncatula*, and *Poa pratensis*, several SWEET transporters are induced under drought stress (Miao et al., 2017; Wang et al., 2018a; Hu et al., 2019; Mathan et al., 2020; Zhang et al., 2020b). In grape berries subjected to a dehydration process aimed to produce raisins, *VvSWEET11* suffered a strong, somewhat unexpected, upregulation (up to 200-fold) 5 d after incubating the bunches at 50°C, while the expression of *VvSWEET15* increased three-fold. Strikingly, after 11 d, when the berries are almost completely dehydrated the expression of *VvSWEET11* remains very high (Conde et al., 2018b). These results suggest that *VvSWEET* may play a role in the redistribution of sugars inside the dehydrated grape tissues (that may behave as osmolytes), but one cannot rule out that the observed overexpression is regulated by temperature, much like it was observed in SWEETs from *Phalaenopsis equestris* (Wang et al., 2018b). In field trials aimed at studying the effect of the application of a protective chemical inert mineral kaolin in grapevines subjected to drought, high irradiance, and high temperatures, *VvSWEET1*, *4*, and *11* were upregulated in treated plants with the “sunscreen”, along with other sugar transporters, as *VvSUC27*. Most likely, kaolin application stimulates sugar transport capacity within the leaves improving source-to-sink transport of sucrose mediated by SWEET (Conde et al., 2018a).

Also, in Arabidopsis, *AtSWEET15* is highly expressed under cold and high salinity (Quirino et al., 1999) and AtSWEET11 and ATSWEET12 are also involved in freezing tolerance. After a cold treatment and under short-day conditions, the double mutant *AtSWEET11/12* accumulates more glucose and fructose and shows higher freezing tolerance than the wild-type (Le Hir et al., 2015). AtSWEET4 also seems to play an important role for plant freezing tolerance. The RNAi4-8 line, with reduced *AtSWEET4* expression accumulated less sugars and showed greater freezing susceptibility, in contrast with *AtSWEET4* overexpression lines, that accumulated more sugars and demonstrated higher freezing tolerance (Liu et al., 2016). In banana, *MaSWEET* genes may play an important role in response to cold, salt, and osmotic stress (Miao et al., 2017) and in *Brassica oleracea* var. *capitata* L. and *P. pratensis* some *SWEET* genes are likely involved in chilling tolerance (Zhang et al., 2019a, 2020b). In tea plant, *CsSWEET1a* and *CsSWEET17* are induced by cold acclimation and cold stress. Accordingly, Arabidopsis plants heterologously expressing these transporters showed higher cold tolerance, further supporting a protective role to CsSWEET1a and 17 during freezing stress (Yao et al., 2020).

The regulation of sugar transport across the vacuolar membrane plays important role in plant response to different environmental stresses. In tea plant, the tonoplast sugar transporter CsSWEET16 is repressed during cold-acclimation, and in Arabidopsis plants overexpressing *CsSWEET16* an increased tolerance to cold was observed, which coincided with the accumulation in the vacuole of glucose and a reduction of fructose (Wang et al., 2018a). Likewise, *AtSWEET16* is repressed in Arabidopsis during cold and osmotic stresses and under cold stress, mutant lines of Arabidopsis overexpressing AtSWEET16 were unable to accumulate fructose, and remarkably, they showed improved germination and increased freezing tolerance (Klemens et al., 2013).

## Conclusion, future perspectives, and intriguing new questions

Plant SWEET transporters and respective physiological roles is currently a hot topic in the plant biology scientific community as demonstrated by the amount of new reports published over the past two years (Abelenda et al., 2019; An et al., 2019; Cai et al., 2019; Doidy et al., 2019; Gautam et al., 2019; Ho et al., 2019; Hu et al., 2019; Liu et al., 2019; Meteier et al., 2019; Wang et al., 2019a, 2019b, 2020; Xie et al., 2019; Zhang et al., 2019b, 2020a, 2020b; Andrés et al., 2020; Breia et al., 2020b; Geng et al., 2020; Li et al., 2020; Mathan et al., 2020; Morii et al., 2020; Ni et al., 2020; Ren et al., 2020; Yao et al., 2020), highlighting both the relevance and rapidly changing knowledge on the topic (see Outstanding Questions).

During the infection process, pathogens are capable of inducing profound metabolic and transcriptomic modifications on their host. Sugar metabolism and mobilization are greatly affected during the infection process and SWEET transporters are important players during this clash. The induction of plant SWEET transporters by pathogens has been linked with an increased capacity of pathogens to obtain host-derived sugars for nutrition. However, plant response to infection may involve SWEET repression to promote sugar starvation of the invading pathogens. SWEET repression may be also induced by pathogens to suppress sugar translocation, which can disrupt various signaling defense pathways. While it is widely accepted that sugar metabolism and mobilization are important players that decide the fate of the ongoing battle between plant and pathogen during the infection process, the metabolic signatures defining the susceptibility or resistance responses of a plant and their regulatory modes, remain poorly understood. There is therefore a need to continuously pay attention to this topic in future research. The challenge to further understand structure/function relationships is also enormous but will bring new insights on the molecular basis of the substrate plasticity and kinetics and energetics properties of SWEETS. Advances driven by physiology, genetics, and biophysics over the past 20 years have dramatically improved our understanding of the molecular basis of plant nutrition and how plants respond to stress. In this context, as nicely reviewed by Schroeder et al. (2013) and Jeena et al. (2019), specialized plant membrane transporters, like SWEETs, can also be molecular targets to enhance yields of staple crops, increase nutrient content, and increase resistance to key stresses, including salinity and pathogens.

## Funding

This work was supported by the Fundação para a Ciência e Tecnologia (FCT), under the strategic programmes UID/AGR/04033/2020 and UID/BIA/04050/2020. This work was also supported by FCT and European Funds (FEDER/POCI/COMPETE2020) through the research project “MitiVineDrought—Combining ‘omics’ with molecular, biochemical, and physiological analyses as an integrated effort to validate novel and easy-to-implement drought mitigation strategies in grapevine while reducing water use” with ref. PTDC/BIA-FBT/30341/2017 and ref. POCI-01-0145-FEDER-030341, respectively; through the research project “BerryPlastid—Biosynthesis of secondary compounds in the grape berry: unlocking the role of the plastid” with ref. POCI-01-0145-FEDER-028165 and ref. PTDC/BIA-FBT/28165/2017, respectively; and also through the FCT-funded research project “GrapeInfectomics” (PTDC/ASPHOR/28485/2017). A.C. was supported with a post-doctoral researcher contract/position within the project “MitiVineDrought” (PTDC/BIA-FBT/30341/2017 and POCI-01-0145-FEDER-030341). R.B. was supported by a PhD student grant (PD/BD/113616/2015) under the Doctoral Programme “Agricultural Production Chains—from fork to farm” (PD/00122/2012) funded by FCT. H.B. was supported by a PhD fellowship funded by FCT (SFRH/BD/144638/2019). This work also benefited from the networking activities within the European Union-funded COST Action CA17111 “INTEGRAPE—Data Integration to maximize the power of omics for grapevine improvement”.

*Conflict of interest statement*. None declared.

**OUTSTANDING QUESTIONS**

- As SWEET transporters are bi-directional and have outstanding substrate plasticity (sugar, polyols, gibberellins), what are the mechanisms that regulate their activity?
- In which additional biological processes related with plant health, development, stress response and reproduction are SWEETs involved?
- Are SWEET transporters “friends or foes” during plant-pathogen interaction? Does it depend on the plant?
- Is the ability of some SWEET transporters (e.g. VvSWEET7) to transport more atypical substrates, like polyols, linked to their increasingly evident relevance also in abiotic stress response?
- Is deciphering SWEET behavior in *B. cinerea* infection of grape berries key for the improvement of productivity and for standardizing production of high-quality and unique “Botrytized” wines?

**ADVANCES**

- Besides key roles in phloem-loading, SWEETs also participate in unloading of sugars in sinks, as observed in tomato young leaves.
- During tuber formation the interaction of a FT-like protein with StSWEET11 blocks StSWEET11 transport activity, promote symplastic sucrose transport.
- SWEETs also transport GAs. In Arabidopsis, GA-transporting SWEETs also transport sucrose, whereas in rice and sorghum they transport glucose.
- SWEET transporters are exploited by pathogens, such as *X. oryzae* pv. *oryzae* by increasing *OsSWEET* expression, however, upregulation of *AtSWEET2* in Arabidopsis roots infected with *P. irregulare* leads to an increased resistance to the pathogen, suggesting a defense-related role.
- *MtSWEETb1* is induced in AM fungicontaining cells and its overexpression promoted the growth of intraradical mycelium, suggesting a key role in glucose exchange in the peri-arbuscular membrane.

## Notes

R.B. conceptualized the work, performed the bibliographical research, and wrote the manuscript. A.C. conceptualized the work and wrote and reviewed the manuscript. H.B. designed and did the artwork. A.M.F. completed the writing. H.G. supervised, completed, and reviewed the writing. A.G. supervised, completed, and reviewed the writing.

The author responsible for distribution of materials integral to the findings presented in this article in accordance with the policy described in the Instructions for Author (https://academic.oup.com/plphys/pages/general-instructions) is: Artur Conde (arturconde@bio.uminho.pt).
